# Patterns and Correlations of Hearing Loss Among Adolescents, Adults, and Elderly in Saudi Arabia: A Retrospective Study

**DOI:** 10.7759/cureus.13913

**Published:** 2021-03-16

**Authors:** Mohammed ALqarny, Abdullah M Assiri, Anas Alshehri, Salmah M Alharbi, Eid H Alshahrani, Halimah Alessa, Somayah A Alghubishi

**Affiliations:** 1 Otolaryngology - Head and Neck Surgery, University of Bisha, Bisha, SAU; 2 Otolaryngology - Head and Neck Surgery, Najran University, Najran, SAU; 3 Otolaryngology - Head and Neck Surgery, King Faisal Medical City, Abha, SAU; 4 Otolaryngology - Head and Neck Surgery, Armed Forces Hospital - Southern Region, Khamis Mushait, SAU; 5 Pediatric Surgery, University of Bisha, Bisha, SAU; 6 General Surgery, Prince Mohammed Bin Nasser Hospital, Jazan, SAU; 7 Medicine, Umm Al-Qura University, Al Qunfudhah, SAU

**Keywords:** hearing loss, hearing impairment, kingdom of saudi arabia (ksa), epidemiology

## Abstract

Background

According to the World Health Organization (WHO), hearing loss (HL) has a significant disease burden with a global prevalence as high a 5% with an expected exponential rise in the coming years. HL has medical, social, and psychological implications on one’s health. A significant proportion of HL cases might be the result of preventable conditions, especially among the young.

Aim

To estimate the pattern of HL and its correlates among adolescent, adult, and elderly populations in the southern regions of Saudi Arabia.

Methodology

A retrospective observational study was carried out between May 2018 and April 2019 across four different ENT clinics located across Saudi Arabia. We included all patients who had a clinical diagnosis of conductive, sensorineural, or mixed HL with varying degrees of severity tested via Pure Tone Audiometer (PTA).

Results

We included 332 cases with HL, ages ranged from 14 to 62 years old with a mean age of 45.2 ±12.6 years. HL was bilateral in 72% of the cases and was associated with tinnitus in 43.1% of cases. Sensorineural HL was the predominant pattern of HL among the elderly and conductive pattern was more common among the young. Causes such as infections, congenital causes, and age-related damage were among the key causes of HL.

Conclusion

Our study showed that HL might be more prevalent among the Saudi population than previously reported. Sensorineural HL is most common among the elderly. However, conductive HL following ear infections is the main cause of HL among young. Therefore, there is a need for significant improvement in public health surrounding ear health to prevent reversible causes of HL, especially among the young.

## Introduction

Hearing loss (HL) or hearing impairment represents partial or complete inability to hear. The challenges posed by HL can range from emotional distress to impaired socialization and difficulties in learning. HL is traditionally divided into three types: conductive, sensorineural, and mixed [1]. According to the World Health Organization (WHO), an estimated 5% of the global population (466 million people) have disabling HL. The WHO report estimates that 34 million children have disabling HL and 60% of cases of childhood HL result from preventable causes [2]. The report also predicts that the cases of HL will rise to over 900 million by 2050 [2]. HL may result from genetic causes, birth complications, infectious diseases, chronic ear infections, use of certain drugs, noise exposure, and aging [3,4]. 

HL is a heterogeneous condition with varying distribution and prevalence across different geographical regions [2]. In Saudi Arabia, there has been some research looking into the prevalence, attributes, and risk factors for HL among children [5,6] and specific population groups such as diabetics [7]. However, to the best of our knowledge, there have been no studies looking into the patterns, attributes, and risk factors for HL among the general Saudi population. 

Therefore, this study aims to identify the demographics of the Saudi population suffering from HL, the patterns of HL, and the risk factors for developing this condition. This knowledge will help the clinicians identify the most common causes of HL among the Saudi population. It will also help the clinicians and the public health authorities devise plans to prevent HL among the Saudi population.

## Materials and methods

Study design

This is a retrospective, observational study that was conducted in four large secondary hospitals in Bisha, Al-Qunfudah, Abha, and Najran. We reviewed the hospital files of patients who attended the ENT clinics in the four cities between May 2018 and April 2019. The study was approved by the research and ethical committee for the college of medicine, University of Bisha, Saudi Arabia (Research and ethical committee approval number 26-018}.

The data was collected from the medical notes of the patients. The collected data included the demographics of the study population, type and side of hearing loss, duration of symptoms, presence of tinnitus, use of hearing aids, and appearance of tympanogram. The hearing assessment was done using a Pure Tone Audiometer (PTA). The severity of hearing loss was determined as per the criteria set by WHO, that is, mild (26-40 dB), moderate (41-60 dB), severe (61-80 dB), and profound (81 dB) [1].

The sample size was calculated using the formula n = (Z)^2^ p (1 - p) / d^2^; where n is the sample size, Z is the Z-score of the confidence interval 95% (set out as 1.96), p represents the estimated population proportion having hearing loss (set out as 0.05 based on the previously mentioned WHO report), and d is the tolerated margin of error (set out as 0.05). The number obtained was 73 cases with confidence level of 95%.

Patient selection (inclusion and exclusion criteria)

Patients who had a clinical diagnosis of conductive, sensorineural, or mixed hearing loss with varying degrees of severity tested via PTA were included in the study. Patients who were not diagnosed with hearing loss, had incomplete information, were younger than 14 years, and patients with pathologies leading to their tinnitus such as Meniere’s disease and vestibular neuronitis were excluded from the study.

Data analysis

The data was collected into an excel spreadsheet that was analyzed using IMB Statistical Package for the Social Sciences (SPSS) for Windows, version 22 (IBM Corp., Armonk, NY, USA). Descriptive analysis based on frequency and percent distribution was done for all demographic and clinical data. Distribution of HL data according to cases bio-demographic characteristics was tested using the Pearson chi-square test. P-value is considered statistically significant at ≤ 0.05. 

## Results

This study included 332 cases with HL. The cases ages ranged between 14 and 62 years old with mean age of 45.2 ±12.6 years (Table 1). 

**Table 1:** Demographics of patients with hearing loss from the four different cities in southern region, Saudi Arabia

Personal data	No (95% CI)	% (95% CI)
Age group		
14-19	58 (45 - 72)	17.5% (13.7 - 21.8%)
20-29	42 (31 - 55)	12.7% (9.4 - 16.5%)
30-39	46 (35 - 59)	13.9% (10.5 - 17.9%)
40-50	42 (31 - 55)	12.7% (9.4 - 16.5%)
>50	144 (127 - 162)	43.4% (38.1 - 48.7%)
Gender		
Male	154 (136 - 172)	46.4% (41.1 - 51.8%)
Female	178 (160 - 196)	53.6% (48.2 - 58.9%)
City		
Al-Qunfudah	43 (32 - 56)	13.0% (9.7 - 16.9%)
Abha	136 (119 - 154)	41.0% (35.8 - 46.3%)
Bisha	84 (69 - 100)	25.3% (20.9 - 30.2%)
Najran	69 (55 - 84)	20.8% (16.7 - 25.4%)

The HL was bilateral in 72% of the cases and it was associated with tinnitus among 43.1% of them. Sensorineural HL was the predominant pattern HL (63.6% cases) and most cases (72.9%) had HL for more than one year. Table 2 describes the patterns of HL among the study population in detail.

**Table 2:** Patterns of hearing loss among patients from the four different cities in southern region, Saudi Arabia

Hearing loss data		No (95% CI)	% (95% CI)
Hearing loss side	Right	58 (45 - 72)	17.5% (13.7 - 21.8%)
	Left	35 (25 - 47)	10.5% (7.6 - 14.2%)
	Bilateral	239 (222 - 254)	72.0% (67.0 - 76.6%)
Hearing loss duration	< 1 year	90 (75 - 106)	27.1% (22.5 - 32.1%)
	> 1 year	242 (226 - 257)	72.9% (67.9 - 77.5%)
Tinnitus	Negative	189 (171 - 206)	56.9% (51.6 - 62.2%)
	Positive	143 (126 - 161)	43.1% (37.8 - 48.4%)
Hearing loss type	Conductive	85 (70 - 101)	25.6% (21.1 - 30.5%)
	Sensorineural	211 (193 - 228)	63.6% (58.3 - 68.6%)
	Mixed	36 (26 - 48)	10.8% (7.8 - 14.5%)
Using hearing aid	Yes	41 (30 - 54)	12.3% (9.1 - 16.2%)
	No	291 (278 - 302)	87.7% (83.8 - 90.9%)
Tinnitus disappeared with hearing aid (n=143)	Yes	19 (12 - 28)	13.3% (8.5 - 19.6%)
	No	124 (115 - 131)	86.7% (80.4 - 91.5%)

**Figure 1:**
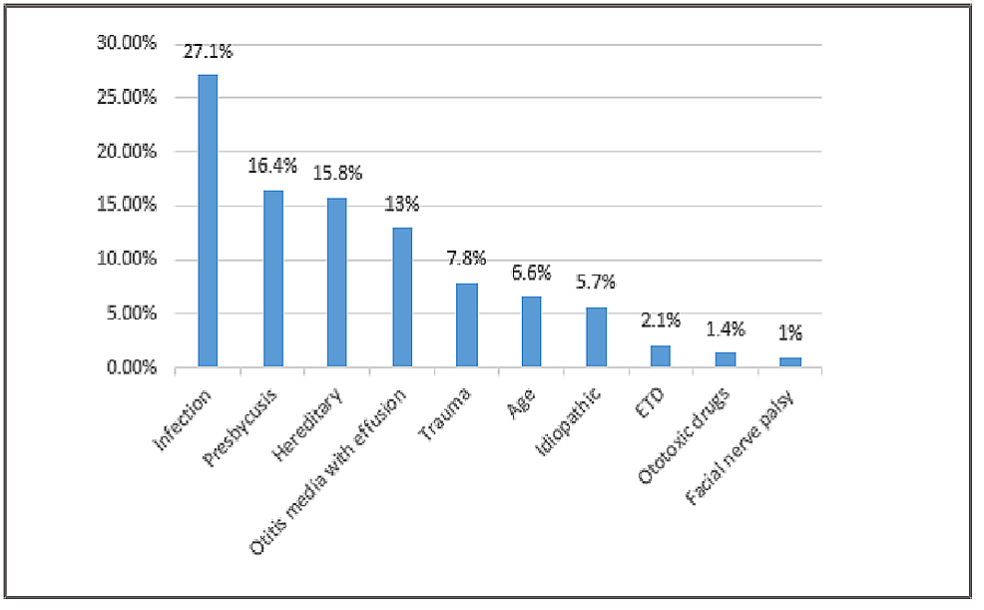
Causes of hearing loss recorded among the Saudi population

Figure 1 shows the main recorded causes of HL among the patients. Infection was the most commonly recorded one (27.1%, 95% CI= 22.4 - 32.5%) followed by presbycusis (16.4%, 95% CI= 12.7 - 21.2%), hereditary HL (15.8%, 95% CI= 12.0 - 20.5%), and otitis media with effusion (13%, 95% CI= 9.9 - 17.8%). While drugs and facial nerve palsy were the least frequently recorded causes.

With regard to Tympanogram findings, Type A was the most recorded (61.1%, 95% CI=56.1 - 66.6%) followed by Type B (33.1%, 95% CI=28.2 - 38.3%), Type C (6%, 95% CI=3.8 - 9.0%), type As (2.1%, 95% CI=1.1 - 4.5%), and type Ad (0.9%, 95% CI=0.2 - 2.8%).

Furthermore, the data analysis suggested that there was a statistical association between age, pattern and type of hearing loss, presence of tinnitus, and appearance on a tympanogram (Table 3). Compared to the adult and younger population, the elderly population was more likely to have bilateral (90.3%) and sensorineural type (85.4%) associated with tinnitus (58.3%). The conductive pattern of hearing loss seemed more prevalent among adolescents (59%) and the young population (47.6%). This is in contrast to other age groups where the conductive pattern of HL was more common (all these factors achieved a statistical significance (P=0.01). As for tympanogram findings, type A pattern seems to be the more predominantly recorded tympanogram pattern among adults (30-49 years) and older population (50+ years) and type B seemed to be the prevalent pattern among the young (20-29) and adolescent (10-19)population (P=.001) (Table 3).

**Table 3:** Distribution of hearing loss according to patients' age P: Pearson X2 test                                * P < 0.05 (significant)

Hearing loss		Age in years								P value
		14-19 years		20-29 years		30-49		50+		
		No	%	No	%	No	%	No	%	
Hearing loss side	Right	8	14.0%	12	28.6%	30	34.1%	8	5.6%	.001*
	Left	6	10.0%	11	26.2%	12	13.6%	6	4.2%	
	Bilateral	44	76.0%	19	45.2%	46	52.3%	130	90.3%	
Tinnitus	Negative	53	91.0%	30	71.4.0%	46	52.3%	60	41.7%	.001*
	Positive	5	9.0%	12	28.6%	42	47.7%	84	58.3%	
Hearing loss type	Conductive	34	59.0%	20	47.6%	22	25.0%	9	6.3%	.001*
	Sensorineural	20	34.0%	20	47.6%	48	54.5%	123	85.4%	
	Mixed	4	7.0%	2	4.84%	18	20.5%	12	8.3%	
Using hearing aid	Yes	7	12.0%	6	14.3%	5	5.7%	23	16.0%	.063
	No	51	82.0%	36	85.7%	83	94.3%	121	84.0%	
Tympanogram	Type A	22	38.0%	18	40.9%	48	54.5%	115	79.9%	.001*
	Type B	35	60.0%	23	52.3%	35	39.8%	17	11.8%	
	Type C	4	7.0%	1	2.3%	7	8.0%	8	5.6%	
	Type As	0	0.0%	1	2.3%	1	1.1%	5	3.5%	
	Type Ad	0	0.0%	1	2.3%	0	0.0%	2	1.4%	

However, there was no statistically significant association between gender and different attributes of hearing loss among the Saudi population (Table 4).

**Table 4:** Distribution of hearing loss according to patients' gender P: Pearson X2 test

Hearing loss		Gender				P
		Male		Female		
		No	%	No	%	
Hearing loss side	Right	23	14.9%	35	19.7%	.211
	Left	13	8.4%	22	12.4%	
	Bilateral	118	76.6%	121	68.0%	
Tinnitus	Negative	88	57.1%	101	56.7%	.941
	Positive	66	42.9%	77	43.3%	
Hearing loss type	Conductive	33	21.4%	52	29.2%	.113
	Sensorineural	107	69.5%	104	58.4%	
	Mixed	14	9.1%	22	12.4%	
Using hearing aid	Yes	18	11.7%	23	12.9%	.733
	No	136	88.3%	155	87.1%	
Tympanogram	Type A	102	66.2%	101	56.7%	.196
	Type B	44	28.6%	66	37.1%	
	Type C	10	6.5%	10	5.6%	
	Type As	2	1.3%	5	2.8%	
	Type Ad	2	1.3%	1	.6%	

## Discussion

Our study found that hearing loss was a common problem among the elderly (50+ years) population (43.4%). Moreover, our study noted that increasing age was significantly associated with sensorineural patterns of hearing loss compared to the conductive pattern of hearing loss seen among the adolescent and young population. About 40% of the cases had associated tinnitus with HL indicating functional problems. Regarding causes of HL, infections were recorded among one-quarter of the cases followed by hereditary disorders and middle ear effusion which is very common among young individuals.

Our finding that hearing impairment is common among the elderly has been consistently reported in the literature. However, in our study, the prevalence of HL among the elderly was as high as 43.4%. Whereas, the prevalence of HL among the elderly in other regional studies is only as high as 37.1% [8]. This apparent discrepancy can be explained by our study design. Unlike the study by Asghari et al. where they divided the elderly population into sub-groups (51-60, 61-70, >71 years), we grouped all the elderly into one group (50+). Our study did not notice any difference between the prevalence and patterns of hearing loss among the two genders, as reported in the literature [8].

Our study also noted that sensorineural hearing loss was the most common pattern of hearing loss followed by the conductive pattern. Further analysis of age groups revealed that sensorineural pattern was more common among the elderly and the conductive pattern was common among adolescents. This is consistent with current literature where the age-related decline in hearing and sensorineural hearing loss is more common in the elderly population [9]. Whereas, the conductive pattern of hearing loss is more commonly reported among adolescents [10].

As for the causes of hearing loss, our study noted that ear infections were the most common cause of hearing loss followed by presbycusis, genetic causes, and middle ear infections. This is consistent with current research where infections, genetic causes, and age-related decline in hearing are among the leading causes of hearing loss among the population [11-16]. The data regarding the pattern and causes of hearing loss provided by our study has important implications. The fact that most of the cases of conductive hearing loss occur among adolescents and are associated with ear infections means that is significant scope for reversibility with appropriate intervention in these cases. Interventions as simple as good ear hygiene and regular check-ups might prove pivotal in preventing the change of reversible hearing loss in irreversible hearing loss. This could help stop HL from affecting school performance and socio-emotional development in 10-15% of school-aged children with mild or slight HL [17-22].

Limitations

However, our study has certain limitations as well. Most of our study population belonged to the elderly age group. This might have led to some degree of age bias in our results. This might also affect the generalization of results to the Saudi population. Furthermore, the convenience-based sampling style of our research might limit the establishment of causality and generalization of results.

## Conclusions

Our study showed that the prevalence of hearing loss among the Saudi population, especially among the elderly population, might be higher than the global and regional average. Furthermore, our study also found that a significant proportion of hearing loss cases, especially among the young, could result from reversible and treatable causes. Therefore, we recommend that larger population-based studies should be carried out to learn more about the prevalence and patterns and hearing loss among the Saudi population, which could guide the development of public health policies leading to better ear health and a reduction in the number of HL cases. Also, Saudi public health should take measures to address preventable causes of HL, especially among the young. 
